# A K-Means Classification and Entropy Pooling Portfolio Strategy for Small and Large Capitalization Cryptocurrencies

**DOI:** 10.3390/e25081208

**Published:** 2023-08-14

**Authors:** Jules Clement Mba, Ehounou Serge Eloge Florentin Angaman

**Affiliations:** School of Economics, College of Business and Economics, University of Johannesburg, Auckland Park, P.O. Box 524, Johannesburg 2006, South Africa

**Keywords:** backtesting, cryptocurrencies, entropy pooling, K-means classification

## Abstract

In this study, we propose three portfolio strategies: allocation based on the normality assumption, the skewed-Student t distribution, and the entropy pooling (EP) method for 14 small- and large-capitalization (cap) cryptocurrencies. We categorize our portfolios into three groups: portfolio 1, consisting of three large-cap cryptocurrencies and four small-cap cryptocurrencies from various K-means classification clusters; and portfolios 2 and 3, consisting of seven small-cap and seven large-cap cryptocurrencies, respectively. Then, we investigate the performance of the proposed strategies on these portfolios by performing a backtest during a crypto market crash. Our backtesting covers April 2022 to October 2022, when many cryptocurrencies experienced significant losses. Our results indicate that the wealth progression under the normality assumption exceeds that of the other two strategies, though they all exhibit losses in terms of final wealth. In addition, we found that portfolio 3 is the best-performing portfolio in terms of wealth progression and performance measures, followed by portfolios 1 and 2, respectively. Hence, our results suggest that investors will benefit from investing in a portfolio consisting of large-cap cryptocurrencies. In other words, it may be safer to invest in large-cap cryptocurrencies than in small-cap cryptocurrencies. Moreover, our results indicate that adding large- and small-cap cryptocurrencies to a portfolio could improve the diversification benefit and risk-adjusted returns. Therefore, while cryptocurrencies may offer potentially high returns and diversification benefits in a portfolio, investors should be aware of the risks and carefully consider their investment objectives and risk tolerance before investing in them.

## 1. Introduction

In recent years, cryptocurrencies have emerged as a new financial asset and are considered potential alternative investments, providing a new perspective for studies on portfolio management. Demiralay et al. [1] define cryptocurrencies as digital currencies that use cryptography to secure and verify transactions. They are based on blockchain technology, a decentralized public ledger that helps record all information (transactions) in a way that makes it difficult to change, hack, or cheat the system. Their primary attribute is that they are not controlled by governments or central banks. The first cryptocurrency, Bitcoin, was created by Satoshi Nakamoto on January 3, 2009, and its success has led to the creation of thousands of other cryptocurrencies, such as Ripple, Ethereum, and Litecoin. As of November 2022, there are approximately 9314 active cryptocurrencies [2].

Lately, the role and importance of cryptocurrencies have grown rapidly, gaining attention among governments, researchers, and investors. Estalayo et al. [3] argue that the introduction and widespread adoption of cryptocurrencies have created a different investment environment where individual can make investment in a personalized, adaptable, and independent manner. Similarly, Lorenzo et al. [4] state that the expansion of cryptocurrencies in terms of number and market capitalization has made the cryptocurrency market a favorable environment for investors, as it offers potential benefits. However, some researchers, such as [5,6], argue that, while cryptocurrencies may have high average returns and a low correlation to traditional assets (stocks and fixed-income securities), these potential benefits come with significant risks that investors must consider carefully before investing in them.

The modern portfolio theory developed by [7] indicates that investors must diversify their portfolio investments by investing in less correlated assets to reduce risk levels and increase portfolio benefits. Since its inception, most empirical studies on modern portfolio theory have focused on stocks and fixed-income securities (see, for example, [8,9,10]). However, the expansion and popularity of cryptocurrencies over the past decade have raised concerns among researchers about whether a traditional investment portfolio would perform better with cryptocurrencies added. As a result, several studies have investigated the diversification benefits of cryptocurrencies in a portfolio of traditional assets (see, for example, [11,12,13]). The majority of those studies found that adding cryptocurrencies to a portfolio of traditional assets outperformed a portfolio of only traditional assets. Therefore, adding cryptocurrencies to a portfolio of traditional assets provides diversification benefits. Additionally, recent research has evaluated the diversification benefits of a portfolio of cryptocurrencies only (see, for instance, [14,15,16]). 

Nevertheless, very few studies have contributed to the literature regarding cryptocurrency pricing and performance analysis. Most of those studies have shown interest in analyzing large- and small-cap cryptocurrencies regarding correlation, shock propagation, stability, and factor structures from a portfolio management perspective. Hence, this study attempts to fill this gap in the portfolio management literature by assessing the performance of large-cap, small-cap, and combined large-small-cap (mixed-cap) cryptocurrencies during crypto market turmoil and under various distribution assumptions.

In this context, the main objective of our study is three-fold: (a) to assess the diversification benefits of combining large-cap and small-cap cryptocurrencies; (b) to evaluate the performance of portfolios relative to distribution assumptions and the incorporation of investors’ views through the EP model; and (c) to backtest the performance of these portfolios during crypto market turmoil. To this end, we employ three portfolio strategies to achieve our objective: allocation based on the normality assumption, the skewed-Student t distribution assumption, and the entropy pooling (EP) method for 14 small- and large-capitalization (cap) cryptocurrencies. We categorize our portfolios into three groups: portfolio 1, consisting of three large-cap and four small-cap cryptocurrencies from various K-means classification clusters; and portfolios 2 and 3, consisting of seven small-cap and seven large-cap cryptocurrencies, respectively. Then, we investigate the performance of the proposed strategies on these portfolios by performing a backtest during a crypto market crash.

The remainder of this paper is organized as follows: Section 2 reviews the literature and highlights our contribution. The methodology is explained in Section 3. Section 4 presents the preliminary data analytics and empirical results. Finally, Section 5 presents the conclusion of the study.

## 2. Literature Review

The modern portfolio theory introduced by [7] suggests that investors must diversify their portfolios by investing across multiple and various assets in order to mitigate risk levels and increase portfolio benefits. Since its introduction, various strategies for constructing portfolios have been developed. The expansion and wide adoption of cryptocurrencies across the world have led some researchers to assess the diversification benefits of cryptocurrencies in portfolios of conventional assets such as stocks and fixed-income securities. For instance, Demiralay et al. [1] use correlation-based conditional diversification benefit measures to analyze the investment benefits of cryptocurrencies using stock prices from eight industrialized nations, eight emerging countries, and eight cryptocurrencies. According to their findings, equity portfolios that include cryptocurrencies provide higher diversification benefits than those that include only stocks. Similarly, Letho et al. [6] employ the daily arithmetic returns of traditional assets (stocks, bonds, currencies), alternative assets (commodities, real estate) in South Africa, and ten cryptocurrencies to evaluate the diversification benefits of cryptocurrencies. They use the mean-variance analysis, Sharpe ratio, conditional value at risk, and mean-variance spanning tests. Their results reveal that adding cryptocurrency to a portfolio of traditional and alternative assets in South Africa produces significant diversification benefits, mainly due to increased portfolio returns. Platanakis et al. [11] investigate the investment benefits of Bitcoin in a traditional portfolio using different asset allocation strategies and find that it generates substantially high risk-adjusted returns. Moreover, Anyfantaki et al. [17] use the stochastic spanning methodology to explore whether including cryptocurrencies in a portfolio of traditional assets (S&P 500, Bond index, and 3-month T-bill) adds value to an investment portfolio. They find that adding cryptocurrency to equity portfolios could benefit risk-loving inestors. However, there do not seem to be any apparent benefits for diversification in adding cryptocurrencies to the portfolios of risk-averse investors. More recently, Tavares et al. [18] investigated the diversification benefits of the top 20 cryptocurrencies on a portfolio of the S&P 100 using volatility timing and reward-to-risk timing. The findings imply that the inclusion of cryptocurrencies in a portfolio of conventional assets does not increase the portfolio’s financial gain. 

K-means clustering has also been used in a few studies to diversify portfolios. For instance, Sharma et al. [19] construct stock portfolios using K-means clustering to reduce risk as per profit. They argue that their study helps to understand the benefit of portfolio diversification to reduce the risk of stock price fluctuations. Rezani et al. [20] study portfolio optimization using iterative K-means -+ and ant colony optimization (ACO) on the Markowitz mean-variance model. Their results show that applying the iterative K-means -+ and ACO methods with eight clusters generates the best risk, average return, and fitness function. Dziuba et al. [21] use K-means clustering to group 30 developed and emerging stock markets using three variables: return, risk, and international diversification. They find that the best risk–return ratio (return 0.46%, risk 5.56%) is from the cluster with an average level of international diversification. Tenkam et al. [22] use the K-means clustering algorithm and the GARCH C-Vine copula model to explore the diversification benefits of a portfolio comprising the 100 largest cryptocurrencies. They find a negative correlation between stablecoins and other cryptocurrencies, implying that they have the potential to be a haven during market instability for cryptocurrency holders.

Another strand of literature investigates the benefits of portfolio diversification among cryptocurrencies. For instance, Dorfleitner et al. [23] analyze the diversification benefits of eight cryptocurrencies using mean-variance spanning tests. They find that seven cryptocurrencies generate significant diversification benefits due to increased portfolio returns, not risk reduction. Yang et al. [16] explore the diversification benefits of the 100 largest cryptocurrencies by market capitalization. They construct efficient and quantile-based sorting portfolios based on ten factors. Their findings show that two price factors can forecast cryptocurrency returns with accuracy. They also find that leverage constraints are essential to controlling the risks associated with cryptocurrency portfolios. In addition, Liu et al. [14] investigates portfolio diversification across 10 major cryptocurrencies and shows that diversification among the cryptocurrencies considerably improves the Sharpe ratio and utility. Rodriguez-Rodriguez et al. [15] employ the entropy measure to construct a portfolio of 18 cryptocurrencies. They find the importance of portfolio diversification as it decreases return uncertainty.

The literature on cryptocurrency pricing and performance analysis has only few research that have contributed to it. Nguyen et al. [24] explore the effect of short-term momentum in the cryptocurrency market from the perspective of asset pricing and portfolio management. They investigate and compare short-term momentum effects, particularly 3-day, 7-day, and monthly momentum effects, in the cryptocurrency market and find that large-cap cryptocurrencies outperform small-cap cryptocurrencies. Thanh et al. [25] analyze the interconnection of the stability of prominent stablecoins, including Tether (USDT), USD Coin (USDC), Paxos Standard (PAX), True USD (TUSD), and DAI, from November 23, 2019 to April 1, 2021. Their results show that large-cap stablecoins drive their small-cap counterparts. Li et al. [26] investigate potential factor structures in the expected returns of crypto assets. They find that large-cap crypto assets with little volatility and high historical returns tend to perform better the following month. Moreover, Hafner et al. [27] build a connectedness network connecting 39 cryptocurrencies based on mutual contributions to the variances of forecast errors for network transaction (NVT) ratios. They find that NVT connectedness is not related to market capitalization, as there are significant and small-cap cryptocurrencies that propagate large NVT shocks. Liew et al. [28] examine the top 100 cryptocurrency returns from 2015 to early 2018. They find that correlations between large-cap cryptocurrencies are higher than correlations between small-cap cryptocurrencies.

The aforementioned studies examined the correlation, shock propagation, stability, and factor structures of both large- and small-cap cryptocurrencies. However, studies on the performance of these two segments of the cryptocurrency market under different scenarios have only received a limited amount of attention, aside from investigating the impact of the short-term momentum of large-cap and small-cap crypto assets from a portfolio management perspective. Therefore, our study contributes to the literature by assessing the performance of large-cap, small-cap, and combination of large-small-cap (mixed-cap) cryptocurrencies amid crypto market turmoil and under various distribution assumptions.

## 3. Methodology

This section describes the different models used in our study. We used R software (R 4.3.1) for the implementation of the models.

### 3.1. K-Means Clustering Algorithm

Clustering is an unsupervised machine-learning task that finds any groupings or clusters in the data. A cluster represents a group of similar data points based on their relationship to surrounding data points. We can classify clustering algorithms into density-based, distribution-based, centroid-based, and hierarchical-based algorithms. Among these types, centroid-based algorithms are the most commonly used. They separate data points based on multiple centroids in the data, and each data point is assigned to a cluster based on its squared distance from the centroid. K-means clustering belongs to this type and is the most straightforward unsupervised learning algorithm. The term K-means was proposed in 1967 by James MacQueen [29]. But the standard algorithm was first introduced as a pulse-code modulation technique by Stuart Lloyd in 1957. In order to group a dataset into distinct clusters, the K-means algorithm uses a distance measure to compare the similarity between different data points. The initial choice of the number K of the centroids determines the final result. The most common method for choosing the number of clusters is to run K-means with different values of K and calculate the variance of the different clusters. This variance is calculated as follows:

(1) $$\sigma =\sum_{j=1}^{K}\sum_{x_{i}\to c_{j}}d{\left(x_{i},c_{j}\right)}^{2}$$

where $c_{j}$ is the cluster center, $x_{i}$ is the ith observation in the cluster with centroid $c_{j}\;$, and $d\left(x_{i},c_{j}\right)$ is the distance between observation $x_{i}$ and the centroid $c_{j}.$ The K-means algorithm works as follows (Algorithm 1):
**Algorithm 1.** K-means algorithmInput: K the number of clusters to be formed X the training set (m × n data matrix) Output:
Randomly choose K < m points (K rows of the data matrix). These points are the centroids.Assign a cluster to each point (or observation), randomly.Calculate the centroid of each cluster (i.e., the vector of the means of the different variables).For each point, calculate its Euclidean distance with the centroids of each of the clusters.Assign the closest cluster to the object.Calculate the sum of the intra-cluster variability.Repeat steps 3 to 5 until an equilibrium is reached, that is, convergence: no more change in clusters, or stabilization of the sum of the intra-cluster variability.


The algorithm converges under either of the following conditions: a pre-set number of iterations, in which case K-means will perform the iterations and stop regardless of the shape of compound clusters; or stabilization of cluster centers (centroids no longer move or change during iterations).

K-means clustering presents quite a number of advantages: it is simple, flexible, and easy to understand and implement. However, according to [30], the user needs to specify the number of clusters in advance. Moreover, its performance depends on an initial centroid, which does not guarantee an optimal solution [30,31].

### 3.2. Entropy Pooling

Meucci et al. [32] proposed the entropy pooling (EP) method, a generalized Bayesian approach that includes market opinions within a prior risk model tuned to the historical data. The unadjusted model (prior) and subjective opinions are combined in this method to obtain a posterior distribution, which can be applied, for example, to risk management and portfolio optimization.

Following are the steps involved in obtaining the posterior distribution from the EP model:

- We assume that a set of random variables X, which has a priori joint distribution, $F_{H}$, can be used to model the market for C assets. X denotes the securities’ return but is not limited to these market factors.
- We assume that the fitted data follows a particular distribution, and we use Monte Carlo simulation to detrmine simulated values for the market distribution. A matrix H of dimensions (R×C) is then obtained, where C represents the marginal prior distributions and R denotes the simulated results for the market factors. To further indicate that each Monte Carlo draw has an identical probality, we associate the probability $P_{r}$ with each of the outcomes $H_{r}$ and set the R probabilities to be equal to 1/R.

Next, we formulate the views (second input). It is represented by a $\left(L\times 1\right)$ vector of function values $K=z={\left(z_{1}\left(X\right),\ldots ,z_{l}\left(X\right),\ldots ,z_{L}\left(X\right)\right)}^{\prime }$ with joint distribution $f_{k}$, a priori. The functions $z_{l}$, l=1,…,L, can be nonlinear in nature. Hence, we estimate the implied distribution of these views using the market simulations H as follows:

(2) $$U_{r,l}=z_{l}\left(H_{r,1},\ldots ,H_{r,c}\right)$$

where l=1,…,L, and r=1,…,R, such that an $\left(R\times L\right)$ matrix contains the empirical distribution of the views implied by the Monte Carlo simulation for the market.

After creating the two panels H and U, we need to couple them to recall the posterior distribution of the market that complies with the views following three steps:

- We express the views based on a set of linear inequality constraints, $b_{lower}\leq B\bar{p}\leq b_{upper}$, where the probability vector $\bar{p}$ is regarded as the objective variable in the following optimization. We obtain the lower and upper bounds $\left(b_{lower},\;b_{upper}\right)$ and the matrix B from U.
- The next step is to minimize the relative entropy. The (discrete) objective function is defined as
  (3) $$RE\left(\bar{p},p\right)=\overset{R}{\underset{r=1}{\sum }}{\bar{p}}_{r}\left[log\left({\bar{p}}_{r})-\log(p_{r}\right)\right],$$

Thus, under the assumption of perfect foresight, we obtain the probabilities of the posterior distribution by estimating

(4) $$\bar{p}=\underset{b_{lower}\leq B\bar{x}\leq b_{upper}}{\underset{︸}{\arg min}}\;RE(x,p)$$

- In the last step, we find the empirical confidence-weighted posterior distribution ($H,\;p_{c})$ as follows:

(5) $$p_{c}=\left(1-c\right)p+c\bar{p}$$

where the confidence level in the views is indicated by the pooling parameter $c\;\in \;\left[0,\;1\right]$. The level of confidence can be described in a variety of ways, as stated in [33]. However, because of the context of views being taken into account on the market within a prior risk model calibrated to the historical data, we assume full confidence in the posterior in our analysis. 

### 3.3. GARCH Model

The GARCH model was developed by [34]. In this study, we used GARCH (1,1) to model the conditional variance. The mean equation is specified as follows:

(6) $$r_{t}=\mu +\varepsilon_{t}$$

The variance equation is specified as

(7) $$\sigma_{t}^{2}=\omega +\alpha_{1}\varepsilon_{t-1}^{2}+\beta_{1}\sigma_{t-1}^{2}$$

where the parameter $\alpha_{1}$ is the ARCH parameter, and $\beta_{1}$ is the GARCH parameter, and the conditional variance process is positive and stationary if the following conditions hold: *ω* > 0, $\alpha_{1}$ > 0, $\beta_{1}$ > 0, and $\alpha_{1}$ + $\beta_{1}$ < 1.

## 4. Results and Analysis

### 4.1. Data and Descriptive Statistics

In this study, we use daily data for 14 cryptocurrencies’ closing prices extracted from the Yahoo Finance database. We extracted the data from 1 August 2019 to 25 October 2022, which results in 1181 daily observations. We collect the top seven cryptocurrencies whose market capitalization is larger than USD 12 billion, namely Bitcoin (BTC), Ethereum (ETH), USDT, Binance (BNB), Ripple (XRP), Cardano (ADA), and Doge, and seven cryptocurrencies whose market capitalization is less than USD 100 million, including iExec RLC (RLC), XYO, Prometeus (PROM), Powerledger (POWR), Ocean Protocol (OCEAN), Numeraire (NMR), and Winklink (WIN). In order to obtain the return series, the cryptocurrencies’ prices are converted to log returns, as shown below:

(8) $$r_{t}=\left(lnp_{t}-lnp_{t-1}\right)$$

where $r_{t}$ denotes the daily log returns, and $p_{t}$ is the daily closing price.

**Figure 1:**
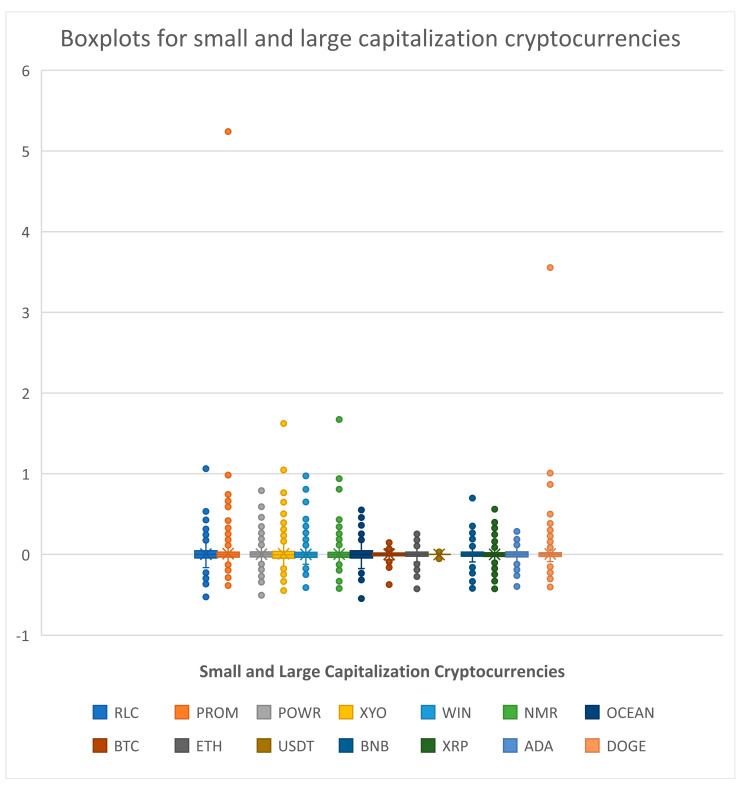
Boxplots for small- and large-capitalization cryptocurrencies.

Table 1 presents the results of the descriptive statistics. We can see that the skewness of most cryptocurrencies’ returns is nonzero, with BTC and ETH being negative. As for the kurtosis, they are all above 3, implying that the empirical distributions of the returns exhibit a fat tail distribution with means around zero. Hence, our series presents the properties of financial time series. We also visualized the data in Table 1 with a box plot (Figure 1). A box plot displays the quartiles of the dataset with points that are determined to be outliers using the interquartile range (IQR). We can observe that most cryptocurrencies in our study present some outliers (points over the maximum and minimum values), except for USDT. This is because USDT is a stablecoin that protects against the volatility of cryptocurrencies.

### 4.2. Empirical Results and Analysis

We can see in Table 2 that the small-cap cryptocurrencies belong to the same cluster except for XYO, PROM, and NMR, which are in individual clusters, respectively. Similarly, the large-cap cryptocurrencies belong to the same cluster except for DOGE and USDT, which are in individual clusters, respectively.

We constructed portfolio 1 by selecting one asset in each cluster to form a portfolio consisting of seven assets, namely USDT, XYO, PROM, OCEAN, NMR, DOGE, and BTC. The reason why we selected OCEAN and BTC in clusters 4 and 7 is that they have the largest market capitalizations in their respective clusters.

As a prior distribution, a skewed-Student t distribution is assumed, and subjective views are formed with respect to the conditional volatilities for each of these cryptocurrency pairs; one-step-ahead forecasts deduced from GARCH (1, 1) models are employed.

We split the entire sample into two sub-periods; our in-sample data contains 1000 observations, and the remaining 181 data points are used for the backtest, where optimal allocations are determined based on the EP posteriors (EP), market distributions (Market), and benchmark allocation, according to a multivariate normal distribution assumption (Normal). The outcome of the three portfolio strategies is presented below against historical performance.

**Figure 2:**
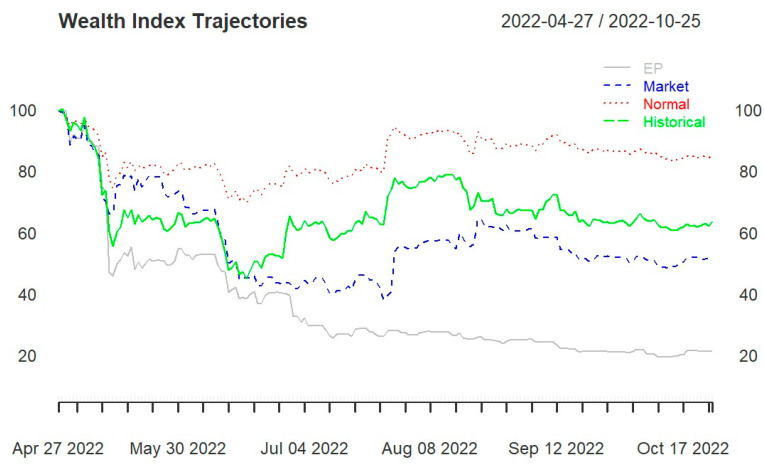
Portfolio 1 wealth trajectories for entropy pooling (EP), market distribution (Market), normal distribution (Normal), and empirical distribution (Historical).

**Figure 3:**
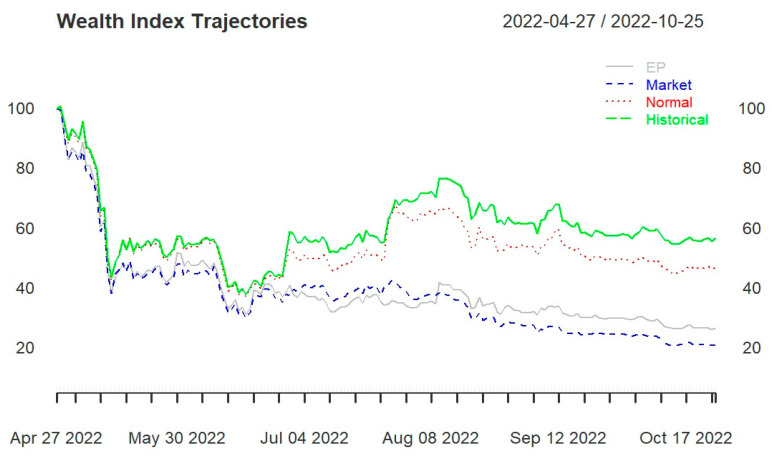
Portfolio 2 wealth trajectories for entropy pooling (EP), market distribution (Market), normal distribution (Normal), and empirical distribution (Historical).

**Figure 4:**
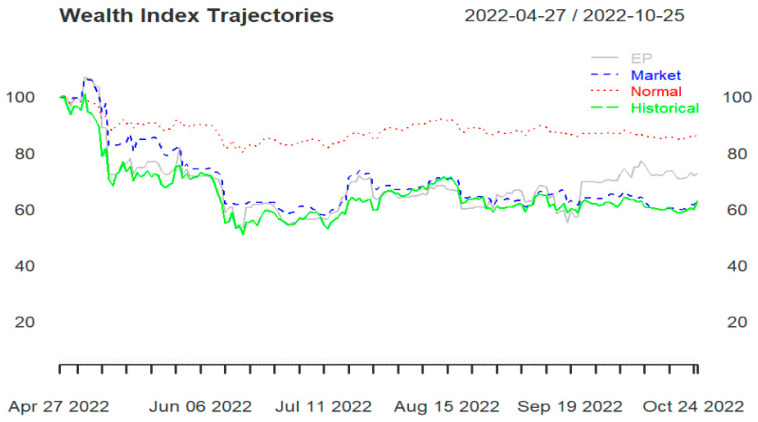
Portfolio 3 wealth trajectories for entropy pooling (EP), market distribution (Market), normal distribution (Normal), and empirical distribution (Historical).

All three portfolios (portfolios 1, 2, and 3) end in losses, regardless of the portfolio strategy used. Starting with a wealth of USD 100, they end below USD 90. Portfolio 1 (Figure 2) and portfolio 3 (Figure 4) are the ones that end at USD 90 under the normality assumption. Portfolio 2 (Figure 3) ends far below USD 60, with the three portfolio strategies performing below the actual historical performance.

Figure 2 and Figure 3 display a sharp drop at the beginning of the backtesting period before stabilizing from mid-May 2022. This may be the result of the shock wave from the hack of the Ronin network, which resulted in USD 625 million being stolen. From the wealth progression in Figure 2, the benchmark allocation based on the normality assumption outperforms the other two portfolio strategies (EP and skewed-Student t distribution) and the actual historical performance. The wealth progression for the EP is the lowest throughout the backtesting period. Note that the subjective views for the EP are formed with respect to the conditional volatilities for each of these cryptocurrency pairs. Pfaff [35] applied the EP model to selected currencies: the weekly euro reference rates in connection with Australian, Canadian, Hong Kong, and US dollars are used, as well as the spot exchange rates of Switzerland and Japan vis-à-vis the euro (Wednesday settlements). The author finds that the allocations according to the EP model regarding the final wealth values achieved exceed those based on the market or normal distributions.

Let us emphasize that 2022 was a dreadful year for crypto investors, though crypto industry sentiment was upbeat at the beginning of 2022 after Bitcoin and Ethereum hit all-time highs in 2021. It was not until March 2022 that the year’s first significant glitch shook the crypto market with the hack of the Ronin network, which supports the popular Axie Infinity blockchain gaming platform, and USD 625 million was stolen, the most significant cryptocurrency theft to date. Many other major crypto platforms, such as Binance and FTX, were also hacked. This situation has put heavy pressure on the crypto market, which has dropped the price of most cryptocurrencies.

Our backtesting period spans the period from April 2022 to October 2022, during which six of the ten most significant hacks that have ever happened took place.

In portfolio 2 (Figure 3), the allocations based on the three strategies perform lower than the actual historical performance and end below USD 60 regardless of the strategy used, with the allocation based on normal distribution outperforming the two other strategies.

In portfolio 3 (Figure 4), the allocation under the skewed-Student t distribution and the EP method perform closely with the actual historical performance but worse than the allocation under the normality assumption. Moreover, portfolio 3 ends with wealth just above USD 60 for all three strategies considered.

Figure 3 and Figure 4 reveal that it may be safer to invest in large-cap cryptocurrencies than in small-cap cryptocurrencies. Although small-cap cryptocurrencies sometimes have a lot of potential (short-term) growth, they are often extremely volatile, considered a risky investment and could crash from one minute to the next.

Aiming at a diversified portfolio, portfolio 1 was formed and could produce similar wealth to portfolio 3 only under the normality assumption strategy (see Figure 2 and Figure 4). In portfolio 3, the skewed-Student t seems to capture well the distribution of data, and the allocations based on EP are close to the actual historical performance.

Therefore, the choice of distribution for the data can significantly impact portfolio performance because it affects the assumptions and calculations used in portfolio optimization and risk management. Though it is well known that financial returns do not follow a normal distribution and instead exhibit fat tails, skewness, and kurtosis, surprisingly, we observed that during certain critical market conditions, portfolios could perform better under normality assumptions, outperforming the skewed-Student t distribution. So, it is essential for investors and portfolio managers to carefully consider the distributional characteristics of financial returns when making investment decisions and modeling portfolio risk.

### 4.3. Performance Measure Analysis

We compute a range of widely used performance measures such as the standard deviation (SD), Sharpe ratio, conditional value at risk (CVaR), and maximum drawdown for our three portfolios. The performance measures assist investors in comparing investments or portfolios on an equal risk basis, enabling them to make well-informed decisions that align with their risk preferences. Table 3, Table 4 and Table 5 report the performance measures for portfolios 1, 2, and 3, respectively, for the entire sample. We can notice that portfolio 3 outperforms the other two portfolios in all performance measures, implying that investors will benefit from investing in a portfolio composed of large-cap cryptocurrencies. In other words, it may be safer to invest in large-cap cryptocurrencies than in small-cap cryptocurrencies. In addition, we find that the second best-performing portfolio is portfolio 1, suggesting that adding large-cap and small-cap cryptocurrencies to a portfolio exhibits some diversification benefits and risk-adjusted returns to investors compared to only investing in a portfolio of small-cap cryptocurrencies like portfolio 2. Our results corroborate our previous findings, where portfolio 3 was found to be the best-performing portfolio, followed by portfolios 1 and 2, respectively.

### 4.4. Sensitivity Test

**Figure 5:**
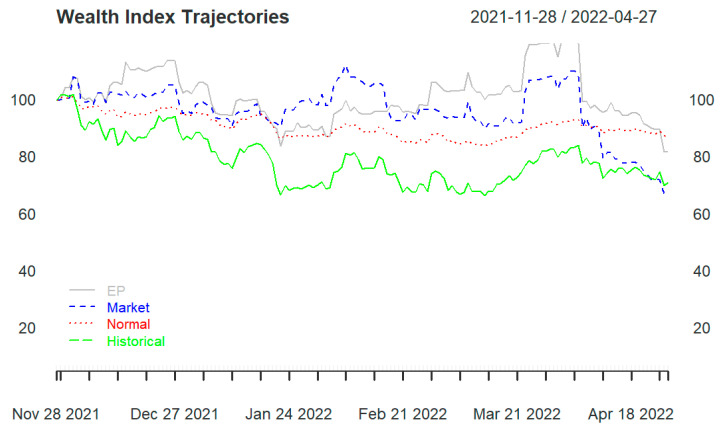
Portfolio 3 wealth trajectory prior to third quarter of April 2022.

**Figure 6:**
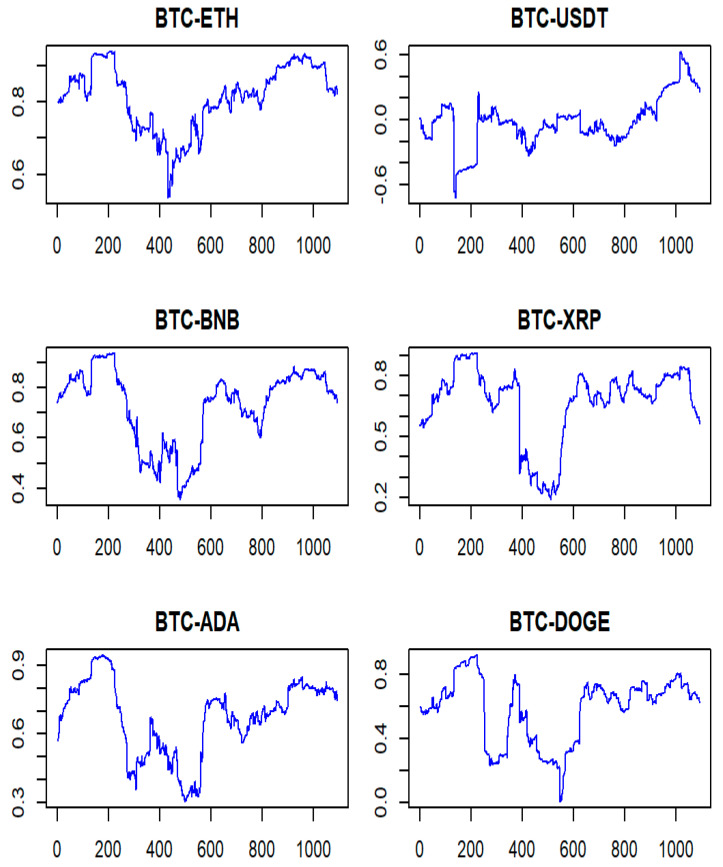
Time-varying correlation of Bitcoin and other cryptos in portfolio 3. Note: The numbers on the horizontal axis represent the number of days.

To further assess the robustness of our results, a backtest was conducted over a period spanning from November 2021 to April 2022. It is important to note that cryptocurrency prices started plummeting in April 2022. Table 1 illustrates that the distribution of the crypto assets used in this study is far from normal based on skewness and kurtosis. Figure 5 displays the wealth trajectory of portfolio 3 and shows that the EP method and the skewed-Student t distribution outperform the approach based on a normal distribution for the period prior to April 2022. Contrasting this result with the findings in Figure 4 illustrates that the distribution of the return series of these crypto assets changes as market conditions change. It may suggest that the crypto market, known for its highly volatile features, i.e., more extreme values than expected, may be well captured by a normal distribution during a period of massive selloff where prices are plummeting, as was the case post-March 2022. During this period, the crypto assets were highly correlated, with an average correlation coefficient of 0.8 as displayed in Figure 6. These correlations were computed with a rolling window of 90 days, which allows a distinctive display of the various changing stages of the correlations.

However, some assets may instead have returns that are approximately normally distributed under stable or normal market conditions. For example, some studies have found that the returns of U.S. Treasury bills are close to normality. Similarly, some exchange rates and price indices may follow a normal distribution when there are no major shocks or interventions in the market.

For the returns of U.S. Treasury bills, Doeswijk et al. [36] create an annual return index for the invested global multi-asset market portfolio. They find that Treasury bills had a compounded annual return of 0.05 and a standard deviation of 0.03 from 1960 to 2017. They also report that Treasury bills have a skewness of −0.12 and a kurtosis of 2.77, which are close to zero and three, respectively, indicating a near-normal distribution.

As for the exchange rates and price indices, Ibbotson et al. [37] construct a global return index of wealth for the period 1960–1984. They use data from 16 countries and include exchange rates and price indices as part of their asset classes. They find that exchange rates have a mean return of 0.00 and a standard deviation of 0.09, while price indices have a mean return of 0.07 and a standard deviation of 0.11. They also report that both asset classes have skewness and kurtosis values that are close to those of a normal distribution.

Some other assets may also have returns that are approximately normally distributed under certain conditions, such as efficient markets, large sample sizes, and short time intervals. Therefore, it is important to properly evaluate or test the distribution features of the return series before applying any statistical methods or models to them. Also, market timing is very important when managing an investment portfolio. As displayed in Figure 5, the beginning of April 2022 sees the performance of the model based on EP and skewed Student t dropping significantly and being taken over by the normality-based approach during the second half of April 2022.

## 5. Conclusions

In this study, we propose three portfolio strategies, namely allocation based on the normality assumption, the skewed-Student t distribution, and the entropy pooling (EP) model, for 14 small- and large-capitalization (cap) cryptocurrencies. Our portfolios are classified into three groups: portfolio 1, composed of three large-cap cryptocurrencies and four small-cap cryptocurrencies from various K-means classification clusters; and portfolios 2 and 3, consisting of seven small-cap and seven large-cap cryptocurrencies, respectively. Then, we examine the performance of these strategies on our portfolios and backtest their performance during a crypto market crash period. Our findings reveal that allocation based on the normality assumption provides better wealth conservation during such a critical period than the other two strategies. We know that the hypothesis that financial variables are normally distributed is often rejected in theoretical studies and particular cases. However, Costa et al. [38] argue that in the “real” world of financial investors, where risk-averse agents mainly hold government bonds and a few equities and do not hold derivatives, the normal distribution still plays a lead role. Hence, the normal distribution assumption outperforming the skewed-Student t distribution assumption in this study should not be surprising. Using a similar approach to FX returns, Pfaff [35] realizes that the allocations based on the skewed-Student t distribution outperform those based on the normal distribution assumption. The reverse is observed in our study. This situation may suggest that the distribution of the financial returns is not static; it changes over time with respect to normal, uptime, and downtime market conditions. Hence, future studies may consider backtesting the performance of the portfolios during uptime and normal market conditions. 

In terms of wealth progression and performance measures, among the three portfolios, portfolio 3, composed of large-cap cryptocurrencies, appears to be the best one, followed by portfolio 1, consisting of a combination of large-cap and small-cap cryptocurrencies. Thus, our results suggest that investors will benefit from investing in a portfolio consisting of large-cap cryptocurrencies. In addition, our results indicate that including large- and small-cap cryptocurrencies in a portfolio may improve diversification and risk-adjusted returns. This situation was illustrated in portfolio 1 compared to portfolio 2 of only small-cap cryptocurrencies, placing portfolio 1 between portfolio 3 and portfolio 2 in order of performance. Though small-cap cryptocurrencies often offer short-term growth potential, they may be potentially highly risky.

Future research may consider entropy pooling with views on the marginal distribution or joint distribution, as they better capture the characteristics of each asset and result in a better-performing entropy pooling method. Furthermore, it would be interesting to investigate how different distribution assumptions and methods perform under different market conditions and for different segments of cryptocurrencies and explore the potential of other portfolio optimization techniques in the context of cryptocurrency investments.

As always with cryptocurrencies, any crypto investor should thoroughly research and consider all the vital factors involved.

## Figures and Tables

**Table 1:** Descriptive statistics.

	Mean	St Dev	Kurtosis	Skewness	Min	Max
RLC	0.0047	0.0858	23.9136	2.0125	−0.5250	1.0621
PROM	0.0094	0.1822	598.1858	21.2665	−0.3842	5.2411
POWR	0.0036	0.0760	19.7747	1.8677	−0.5036	0.7906
XYO	0.0080	0.1193	39.9834	4.0133	−0.4460	1.6221
WIN	0.0015	0.0787	36.1761	3.4134	−0.4091	0.9737
NMR	0.0047	0.1053	126.3483	8.6648	−0.4203	1.7282
OCEAN	0.0054	0.0851	6.7452	0.7899	−0.5452	0.5510
BTC	0.0013	0.0379	10.2696	−0.5846	−0.3717	0.1875
ETH	0.0029	0.0495	7.4573	−0.4516	−0.4235	0.2595
USDT	0.0000	0.0035	97.9247	0.8768	−0.0512	0.0548
BNB	0.0035	0.0564	26.7490	1.6034	−0.4190	0.6976
XRP	0.0019	0.0614	16.5649	1.3566	−0.4233	0.5601
ADA	0.0035	0.0582	4.6427	0.3358	−0.3957	0.3224
DOGE	0.0071	0.1311	475.3333	18.2215	−0.4026	3.5555

Note: Table 1 reports descriptive statistics of the 14 cryptocurrencies’ returns. RLC, PROM, POWR, XYO, WIN, NMR, and OCEAN represent the small-cap cryptocurrencies, and BTC, ETH, USDT, BNB, XRP, ADA, and DOGE constitute the large-cap cryptocurrencies. The sample period is from August 1, 2019 to October 25, 2022.

**Table 2:** K-means clusters.

Assets	USDT	XYO	PROM	RLC	POWR	WIN	OCEAN
Clusters	1	2	3	4	4	4	4
Assets	NMR	DOGE	BTC	ETH	XRP	BNB	ADA
Clusters	5	6	7	7	7	7	7

Note: Each color represents a cluster. So, in total, we have 7 clusters.

**Table 3:** Performance measures for portfolio 1.

	EP	Market	Normal
Return (annual)	−30.5346	−14.7348	−10.6871
Risk (annual, SD)	26.8966	28.4089	16.8217
Sharpe ratio	−1.13526	−0.51867	−0.63532
CVaR (modified, 95%)	11.63372	10.73807	6.11421
Maximum drawdown	90.37545	74.47341	53.81820

**Table 4:** Performance measures for portfolio 2.

	EP	Market	Normal
Return (annual)	−25.2552	−32.7138	−31.8660
Risk (annual, SD)	36.7911	39.1523	35.9401
Sharpe ratio	−0.68644	−0.83555	−0.88664
CVaR (modified, 95%)	11.78649	12.59805	11.57225
Maximum drawdown	92.39578	92.53891	91.81616

**Table 5:** Performance measures for portfolio 3.

	EP	Market	Normal
Return (annual)	−20.0126	−22.7509	−5.88515
Risk (annual, SD)	24.3576	20.14378	12.50122
Sharpe ratio	−0.82161	−1.12943	−0.47077
CVaR (modified, 95%)	10.31025	7.814897	4.36737
Maximum drawdown	79.82941	81.53747	37.06805

## Data Availability

The data can be obtained from any of the authors upon request.

## References

[1] Demiralay S., Bayracı S. (2021). Should stock investors include cryptocurrencies in their portfolios after all? Evidence from a conditional diversification benefits measure. Int. J. Financ. Econ..

[2] Cryptocurrency Prices, Charts and Market Capitalizations|CoinMarketCap 2022. https://coinmarketcap.com/.

[3] Estalayo I., Del Ser J., Osaba E., Bilbao M.N., Muhammad K., Gálvez A., Iglesias A. Return, Diversification and Risk in Cryptocurrency Portfolios using Deep Recurrent Neural Networks and Multi-Objective Evolutionary Algorithms. Proceedings of the IEEE Congress on Evolutionary Computation.

[4] Lorenzo L., Arroyo J. (2022). Analysis of the cryptocurrency market using different prototype-based clustering techniques. Financ. Innov..

[5] Kim S., Sarin A., Virdi D. (2018). Crypto-assets unencrypted. J. Investig. Manag..

[6] Letho L., Chelwa G., Alhassan A.L. (2022). Cryptocurrencies and portfolio diversification in an emerging market. China Financ. Rev. Int..

[7] Markowitz H. (1952). Portfolio Selection. J. Financ..

[8] Pfiffelmann M., Roger T., Bourachnikova O. (2016). When behavioral portfolio theory meets Markowitz theory. Econ. Model..

[9] Malkiel B.G. (2019). A Random Walk down Wall Street the Time-Tested Strategy for Successful Investing.

[10] Mangram M.E. (2013). A simplified perspective of the Markowitz portfolio theory. Glob. J. Bus. Res..

[11] Platanakis E., Urquhart A. (2019). Should investors include bitcoin in their portfolios? A portfolio theory approach. Br. Account. Rev..

[12] Lee D.K.C., Guo L., Wang Y. (2017). Cryptocurrency: A new investment opportunity?. J. Altern. Investig..

[13] Briere M., Oosterlinck K., Szafarz A. (2015). Virtual currency, tangible return: Portfolio diversification with bitcoin. J. Asset Manag..

[14] Liu W. (2019). Portfolio Diversification across Cryptocurrencies. Financ. Res. Lett..

[15] Rodriguez-Rodriguez N., Miramontes O. (2022). Shannon Entropy: An Econophysical Approach to Cryptocurrency Portfolios. Entropy.

[16] Yang Y., Zhao Z. (2021). Large cryptocurrency-portfolios: Efficient sorting with leverage constraints. Appl. Econ..

[17] Anyfantaki S., Arvanitis S., Topaloglou N. (2021). Diversification benefits in the cryptocurrency market under mild explosivity. Eur. J. Oper. Res..

[18] Tavares R.D.S., Caldeira J.F., Raimundo Junior G.D.S. (2022). It’s all in the timing again: Simple active portfolio strategies that outperform naïve diversification in the cryptocurrency market. Appl. Econ. Lett..

[19] Sharma R., Arora S. (2022). Risk Reduction by Stock Portfolio Selection using LSTM and K-means Clustering. Int. J. Adv. Res. Sci. Commun. Technol..

[20] Rezani M.A., Hertono G.F., Handari B.D. Implementation of Iterative K-Means-+ and Ant Colony Optimization (ACO) in Portfolio Optimization Problem. Proceedings of the 5th International Symposium on Current Progress in Mathematics and Sciences (ISCPMS2019).

[21] Dziuba P., Glukhova D., Shtogrin K. (2022). Risk, Return and International Portfolio Diversification: K-Means Clustering data. Balt. J. Econ. Stud..

[22] Tenkam H.M., Mba J.C., Mwambi S.M. (2022). Optimization and Diversification of Cryptocurrency Portfolios: A Composite Copula-Based Approach. Appl. Sci..

[23] Dorfleitner G., Lung C. (2018). Cryptocurrencies from the perspective of euro investors: A re-examination of diversification benefits and a new day-of-the-week effect. J. Asset Manag..

[24] Nguyen H., Liu B., Parikh N.Y. (2020). Exploring the short-term momentum effect in the cryptocurrency market. Evol. Institutional Econ. Rev..

[25] Thanh B.N., Hong T.N.V., Pham H., Cong T.N., Anh T.P.T. (2022). Are the stabilities of stablecoins connected?. J. Ind. Bus. Econ..

[26] Li J., Yi G. (2019). Toward a factor structure in crypto asset returns. J. Altern. Investig..

[27] Hafner C.M., Majeri S. (2022). Analysis of cryptocurrency connectedness based on network to transaction volume ratios. Digit. Financ..

[28] Liew J., Li R.Z., Budavári T., Sharma A. (2019). Cryptocurrency investing examined. J. Br. Blockchain Assoc..

[29] Agarwal S., Yadav S., Singh K. Notice of Violation of IEEE Publication Principles: K-means versus K-means++ clustering technique. Proceedings of the 2012 Students Conference on Engineering and Systems.

[30] Serban G., Moldovan G.S. (2006). A comparison of clustering techniques in aspect mining. Informatica.

[31] Shafeeq A., Hareesha K.S. Dynamic clustering of data with modified K-means algorithm. Proceedings of the 2012 Conference on Information and Computer Networks.

[32] Meucci A. (2006). Beyond Black-Litterman in practice: A five-step recipe to input views on non-normal markets. Risk.

[33] Meucci A. (2008). The Black-Litterman Approach: Original Model and Extensions. SSRN Electron. J..

[34] Bollerslev T. (1986). Generalized Autoregressive Conditional Heteroskedasticity. J. Econom..

[35] Pfaff B. (2016). Financial Risk Modelling and Portfolio Optimization with R.

[36] Doeswijk R., Lam T., Swinkels L. (2019). Historical Returns of the Market Portfolio. Rev. Asset Pricing Stud..

[37] Ibbotson R.G., Siegel L.B., Love K.S. (1985). World Wealth. J. Portf. Manag..

[38] Costa M., Cavaliere G., Iezzi S. (2005). The role of the normal distribution in financial markets. New Developments in Classification and Data Analysis: Proceedings of the Meeting of the Classification and Data Analysis Group (CLADAG) of the Italian Statistical Society, University of Bologna, Bologna, Italy, 22–24 September 2003.

